# Synthesis of 2-aminosuberic acid derivatives as components of some histone deacetylase inhibiting cyclic tetrapeptides

**DOI:** 10.3762/bjoc.13.214

**Published:** 2017-10-17

**Authors:** Shital Kumar Chattopadhyay, Suman Sil, Jyoti Prasad Mukherjee

**Affiliations:** 1 Department of Chemistry, University of Kalyani, Kalyani - 741235, West Bengal, India

**Keywords:** α-amino acid, catalysis, chiral pool, cross metathesis, cyclic peptides

## Abstract

A new synthesis of the important amino acid 2-aminosuberic acid from aspartic acid is reported. The methodology involves the alternate preparation of (*S*)-2-aminohept-6-enoate ester as a building block and its diversification through a cross-metathesis reaction to prepare the title compounds. The utility of the protocol is demonstrated through the preparation of three suberic acid derivatives of relevance to the design and the synthesis of peptides of biological relevance.

## Introduction

**Figure 1:**
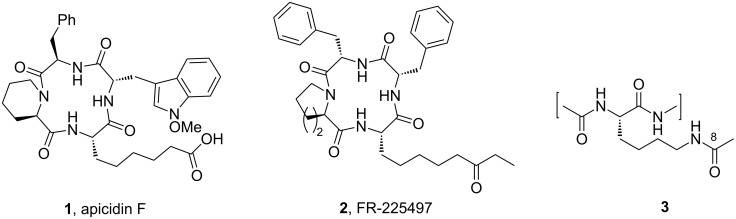
Biologically active naturally occurring cyclic tetrapeptide HDAC inhibitors.

α-Aminosuberic acid (Asu) is a component of apicidin F (**1**, Figure 1) belonging to an interesting class of cyclic tetrapeptides displaying antimalarial and histone deacetylase inhibitory (HDACi) properties [1–2]. It has been suggested [3] that the terminal carbonyl group in members of this family (e.g., in **2**) functionally mimics the C-8 keto group of the acetylated lysine residue (**3**) of histones as a part of their biological activity and therefore the variation in the carbonyl functionality may have implications in drug design. Moreover, Asu and its congener 2-aminopimelic acid have been used as ethylenic equivalent of a disulfide linkage [4]. Other applications of Asu in peptide engineering and as a building block are of notable importance [5–6]. For this, and other reasons, several synthetic routes to Asu have been developed [7–9] which often uses chemical or enzymatic resolution of a racemate [10]. However, the chemical synthesis of an orthogonally protected Asu derivative from easily available sources [11] for potential applications remains important. During the course of our work on the synthesis of amino acids and peptides relevant to HDAC inhibition [12], we required orthogonally protected Asu derivatives. Herein we describe an alternate synthesis of the important building block 2-aminoheptenoic acid and its application to the synthesis of orthogonally protected Asu derivatives.

## Results and Discussion

**Scheme 1:**
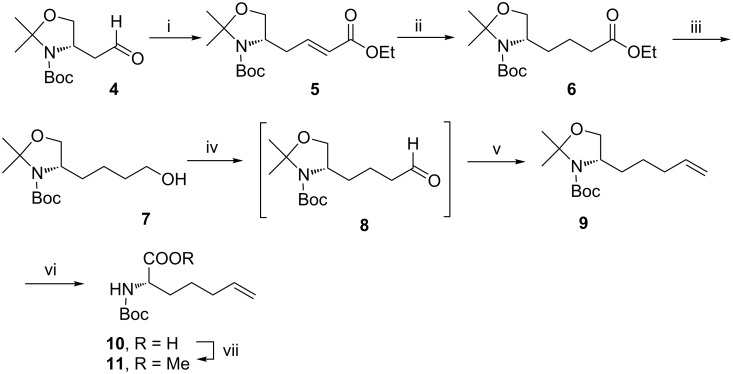
Reagents and conditions: (i) Triethyl phosphonoacetate, *n*-Bu_4_N^+^I^−^, aq K_2_CO_3_, rt, 18 h, 86%; (ii) H_2_, Pd/C, EtOAc, rt, 6 h, 83%; (iii) LAH, THF, 0 °C to rt, 2 h, 81%; (iv) (COCl)_2_, DMSO, *N*-methylmorpholine, CH_2_Cl_2_, −78 °C to 0 °C; (v) MePh_3_PBr, *n*-BuLi, THF, 0 °C, 3 h, 72% over two steps; (vi) chromic acid, acetone, 2 h, 73%; (vii) Cs_2_CO_3_, CH_3_I, DMF, 2 h, 88%.

Our synthesis started with the preparation of bishomoallylglycine derivative **11** (Scheme 1) from the known aspartic acid derived aldehyde **4** [13]. The latter was converted to its doubly homologated derivative **8** through four conventional steps viz. HWE-type olefination leading to the unsaturated ester **5**, saturation of the double bond in the latter to **6**, reduction of the ester moiety in **6** to the alcohol **7** followed by its oxidation. The aldehyde **8** thus obtained was subjected to a Wittig olefination to obtain the desired alkene **9** in an overall yield of 42% over five steps. One-pot deprotection–oxidation [14] of the oxazolidine moiety in **9** proceeded uneventfully to provide *N*-Boc-2-amino-6-heptenoic acid (**10**) in good yield. The latter was smothly protected as its methyl ester using methyl iodide in the presence of cesium carbonate to provide the desired 2-aminoheptenoic acid derivative **11**. Several syntheses of this important amino acid have appeared which include Lubell’s palladium-catalyzed allylation [15], Riera’s asymmetric epoxidation protocol [16], Rich’s enolate amination [17] and Hruby’s asymmetric alkylation [18] of a chiral nickel complex among others. Moreover, many of these studies have also reported an elegant use of this unsaturated amino acid [19]. Our simple protocol involves the use of less sophisticated reagents and catalysts and the use of easily available starting materials; it proceeds in an overall yield of 27% over seven high yielding simple steps.

**Scheme 2:**
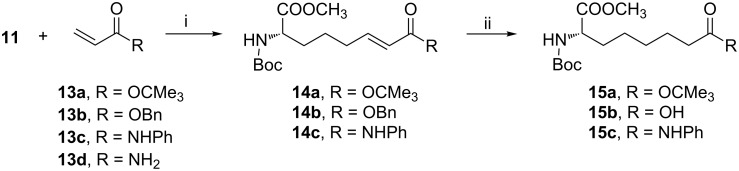
Reagents and conditions: (i) Grubbs’ catalyst **12** (2.5 mol %), DCM, reflux, 2 h, **14a**, 83%; **14b**, 90%; **14c**, 88% ; (ii) H_2_, Pd/C, MeOH, rt, 2 h, **15a**, 79%; **15b**, 82%; **15c**, 90%.

Having access to the building block **11**, we focused on its conversion to the targeted Asu derivatives through cross metathesis (CM) [20] with conjugated olefins **13a–d** (Scheme 2). In recent years, the cross-metathesis reaction has emerged as a valuable tool in the preparation of α-amino acids [21–27] and few useful general guidelines have emerged from these studies. Pleasingly, cross metathesis of our building block **11** with *tert*-butyl acrylate (**13a**) proceeded quickly in the presence of Grubbs’ 2nd generation catalyst [(1,3-bis(2,4,6-trimethylphenyl)-2-imidazolidinylidene)dichloro(phenylmethylene)(trichlorohexylphosphine)ruthenium, **12**] in refluxing dichloromethane and the product **14a** was obtained in good yield. The corresponding reaction of **11** with benzyl acrylate (**13b**) proceeded with similar facility, yield and isomeric composition. Although the cross-metathesis reaction with α,ß-unsaturated esters and ketones have been extensively studied, the corresponding reactions with amides and anilides are less documented [28]. An elegant solution to one of this problems is the use of acryloyl chloride as CM partner followed by functionalization of the cross product [29]. To our delight, the reaction of **11** with anilide **13c** proceeded well under our developed conditions and the CM product was obtained as a single isomer (*E*-). Only a few successful reports on cross metathesis with Weinreb’s amide of acrylic acid, and N-alkylated acrylamides are known [30–33]. However, all attempts of CM reaction of **11** with the olefin **13d** proved to be futile, a major problem being the inadequate solubility of the olefin in the reaction solvents tried, e.g., dichloromethane, dichloroethane, benzene, toluene etc. The solubility problem may be avoided by dilution and increasing the temperature but the reaction is too slow to be useful. The CM product **14a** was then hydrogenated to obtain the known Asu derivative **15a** [34] under conventional conditions. Similarly, the known *N*-Boc-L-Asu-OH (**15b**) [35] was obtained by hydrogenation of the benzyl ester **14b** with concomitant saturation of the double bond. The conversion of **14c** into **15c** proceeded without events.

## Conclusion

In conclusion, we have developed a modestly diversified synthesis of important Asu derivatives through an alternate preparation of the building block **11** of proven utility in the design and synthesis of peptidomimetics. A cross-metathesis reaction has been utilized to create the diversification on the template **11** in order to obtain orthogonally protected Asu derivatives. Moreover, the Asu derivative **15a** has been demonstrated to be useful in the preparation of a plethora of HDAC inhibitors [34]. The methodology may therefore find application in the synthesis of related targets and may complement to the existing literature. The work will be continued to explore syntheses of other Asu derivatives using the developed methodology.

## Experimental

### General procedure for cross metathesis

This was carried out in a manner as described in [36]. Grubb’s second generation catalyst **12** (10 mg, 0.012 mmol, 2.5 mol %) was added to a stirring solution of olefin **11** (130 mg, 0.50 mmol) in dry DCM (1 mL) and then a solution of the appropriate electron-deficient olefin **13** (1.5 mmol) in dry DCM (1 mL) was added dropwise under an argon atmosphere. The resulting reaction mixture was then heated to reflux for 2 h. The reaction mixture was allowed to cool to room temperature and then concentrated in vacuo. The residue was subjected to column chromatographic purification over silica gel using an appropriate mixture of ethyl acetate in hexane to provide the coupled product as colorless viscous liquid.

### (*S,E*)-1-*tert*-Butyl 8-methyl 7-(*tert*-butoxycarbonylamino)oct-2-enedioate (**14a**)

Colourless liquid. Yield: 148 mg, 83%; [α]_D_^25^ +12.60 (*c* 1.00, CHCl_3_); IR (neat): 3363, 2978, 2933, 1715, 1652, 1505, 1367, 1164 cm^−1^; ^1^H NMR (400 MHz, CDCl_3_) δ 6.81 (td, *J* = 6.8, 15.6 Hz, 1H), 5.74 (d, *J* = 15.6 Hz, 1H), 5.10 (d, *J* = 7.6 Hz, 1H), 4.31 (m, 1H), 3.74 (s, 3H), 2.23–2.17 (m, 2H), 1.81 (m, 1H), 1.65 (m, 1H), 1.58–1.48 (m, 11H), 1.44 (s, 9H) ppm; ^13^C NMR (100 MHz, CDCl_3_) δ 173.2, 165.9, 155.3, 146.7, 123.6, 80.1, 79.9, 53.1, 52.3, 32.2, 31.4, 28.3, 28.1, 23.8 ppm; HRMS (TOF–MS ES^+^) *m/z*: [M + Na]^+^ calcd for C_18_H_31_NNaO_6_, 380.2049; found, 380.2056.

## Supporting Information

File 1 Experimental details and analytical data of all new compounds as well as copies of their ^1^H and ^13^C NMR spectra.
