# Supplementary material for: Synthesis of 2-aminosuberic acid derivatives as components of some histone deacetylase inhibiting cyclic tetrapeptides
Source: Beilstein J Org Chem. 2017 Oct 17;13:2153–6. doi: 10.3762/bjoc.13.214 (PMC5669242; doi:10.3762/bjoc.13.214)
Supplement: File 1 — Experimental details and analytical data of all new compounds as well as copies of their 1H and 13C NMR spectra. [file Beilstein_J_Org_Chem-13-2153-s001.pdf]

Supporting Information

for

Synthesis of 2-aminosuberic acid derivatives as components of some histone deacetylase inhibiting cyclic tetrapeptides

Shital Kumar Chattopadhyay*[§] Suman Sil and Jyoti Prasad Mukherjee

Address: Department of Chemistry, University of Kalyani, Kalyani - 741235, West Bengal, India

*Corresponding author

Email: Shital Kumar Chattopadhyay - skchatto@yahoo.com

[§]Fax: +91+33+25828282

Experimental details and analytical data of all new compounds as well as copies of their ^{1}H and ^{13}C NMR spectra

Content **page**

Experimental procedure ……………………………………………………………………….S2–S10

Copies of ^{1}H and ^{13}C NMR spectra of the reported compounds -------------------------------- S11–S32

Experimental

General: Column chromatography was performed on silica gel, Merck grade 230–400 mesh and neutral alumina. Reactions were monitored by thin-layer chromatography; TLC plates were visualized with UV, in an iodine chamber, or with vaniline solution, unless noted otherwise. Melting points were recorded in open capillaries and are uncorrected. IR spectra were recorded using KBr disks, chloroform solution or as neat. Chemical shifts (δ) are given from TMS (0 ppm) as internal standard for ^{1}H NMR and $^{13}CDCl_3$ (77.0 ppm) for ^{13}C-NMR. The following abbreviations were used to explain the multiplicities: s = singlet, d = doublet, t = triplet, q = quartet, m = multiplet, dd = double doublet, ddd = doublet of double doublet, dt = doublet of triplet, br = broad, etc. The data in the parentheses indicate signals due to rotamerism. HRMS data were recorded on a Waters XEVO G2 QTOF instrument purchased through DST-PURSE Grant.

Dichloromethane and dimethyl sulfoxide were distilled over calcium hydride under an inert atmosphere. THF, toluene, benzene and ether were freshly distilled under argon from a purple solution of sodium benzophenone ketyl. Unless stated otherwise, all reagents were purchased from commercial sources and used without additional purification.

(*S*,*E*)-*tert*-Butyl 4-(4-ethoxy-4-oxobut-2-enyl)-2,2-dimethyloxazolidine-3-carboxylate (5)

Triethyl phosphonoacetate (3.6 mL, 17.65 mmol) was added to a stirring solution of the aldehyde **4** (3.58 g, 14.71 mmol) followed by the addition of tetrabutylammonium iodide (0.55 g, 10 mol%) and aqueous K_2CO_3 solution (3.0 M, 5.9 mL). The resulting mixture was vigorously stirred at rt for 18 h. It was then diluted with water (50 mL) and extracted with ether (2 × 50 mL). The combined organic layer was washed successively with water (2 × 75 mL) and brine (75 mL). It was then dried over anhydrous Na_2SO_4, filtered and the filtrate was concentrated under reduced pressure to leave a crude product which on column chromatography on silica gel using EtOAc in petroleum ether (1:49) provided the α, β-unsaturated ester **5** as a colorless liquid.

Yield: 3.96 g, 86%.

$[\alpha]_D^{25}$ = +26.7 (*c* 2.5, $CHCl_3$);

IR ($CHCl_3$) 3380, 2982, 2938, 2878, 1722, 1698, 1655, 1389 cm^{-1};

^{1}H NMR (400 MHz, $CDCl_3$) δ = 6.82 (m, 1 H), 5.79 (d, *J* = 15.6 Hz, 1 H), 4.11-3.99 (m, 3 H), 3.87 (m, 1 H), 3.66 (m, 1 H), 2.58-2.36 (m, 2 H), 1.57-1.45 (m, 3 H), 1.40 (s, 12 H), 1.21-1.47 (m, 3 H).

^{13}C NMR (100 MHz, $CDCl_3$): δ = 166.2, 152.0 (151.5), 144.6, 123.8 (123.7), 94.1 (93.5), 80.3 (79.9), 66.8 (66.2), 60.3 (60.2), 56.2 (56.1), 36.9 (35.6), 28.4, 27.4 (26.8), 24.4 (23.1), 14.2.

HRMS (QTOF ES+): *m/z* $[M + Na]^+$ calcd for $C_{16}H_{27}NNaO_5$:336.1787; found: 336.1781.

(*S*)-*tert*-Butyl 4-(4-ethoxy-4-oxobutyl)-2,2-dimethyl-oxazolidine-3-carboxylate (6)

This was prepared in a manner similar as described in [27]. Palladium on charcoal (150 mg) was added portion wise to a stirring solution of the compound **5** (2.5 g, 7.94 mmol) in ethyl acetate (15 mL) at room temperature and the heterogeneous solution was stirred under hydrogen atmosphere for 6 h. It was then filtered through Celite and the filter cake was thoroughly washed with ethyl acetate (30 mL). The combined filtrate was concentrated in vacuo to furnish the product **6** as a colorless liquid.

Yield: 2.08 g, 83%.

$[\alpha]_D^{25}$ = +21.6 (*c* 0.75, $CHCl_3$)

IR ($CHCl_3$) 3381, 2982, 2936, 2873, 1722, 1698, 1653, 1389 cm^{-1}

^{1}H NMR (400 MHz, $CDCl_3$) δ = 4.12-3.97 (m, 2 H), 3.93-3.91 (m, 2 H), 3.74 (m, 1H), 2.33-2.31 (m, 2 H), 1.63-1.52 (m, 7 H), 1.47 (s, 12 H), 1.27-1.25 (m, 3 H).

^{13}C NMR (100 MHz, $CDCl_3$): δ = 172.9, 151.9 (151.5), 93.4 (92.9), 79.7 (79.2), 66.8 (66.5), 60.0, 57.1 (56.8), 33.8, 33.1 (32.2), 28.2, 27.4 (26.6), 24.3 (22.9), 21.4, 14.0.

HRMS (QTOF ES+): *m/z* $[M + Na]^+$ calcd for $C_{16}H_{29}NNaO_5$ requires 338.1943; found: 338.1931.

(*S*)-*tert*-Butyl 4-(4-hydroxybutyl)-2,2-dimethyloxazolidine-3-carboxylate (7)

To a stirred suspension of $LiAlH_4$ (0.673 g, 18.05 mmol) in dry THF (30 mL), a solution of the ester **6** (5.68 g, 18.05 mmol) in THF (20 mL) was added drop wise at 0 °C under nitrogen atmosphere. It was stirred for 2 h at room temperature and then quenched under ice-cold condition with saturated KOH solution. The reaction mixture was then filtered through celite and the filtrate was concentrated under reduced pressure to give colourless crude product which was purified by chromatography over silica using mixture of petroleum ether and ethyl acetate (4:1) as eluent to afford colourless oil **7**.

Yield: 3.99 gm (81%)

$[\alpha]_D^{25}$ = +9.40 (*c* 1.5, $CHCl_3$);

IR ($CHCl_3$) 3446, 2980, 2937, 2869, 1697, 1457, 1392, 1366 cm^{-1};

^{1}H NMR (400 MHz, $CDCl_3$) δ = 3.86-3.74 (m, 1 H + br s, 1 H), 3.68-3.66 (m, 2 H), 3.54 (s, 2 H), 2.95-2.64 (m, 2 H), 1.72 (m, 1 H), 1.51-1.47 (m, 7 H), 1.41 (s, 9 H), 1.30-1.26 (m, 2 H).

^{13}C NMR (100 MHz, $CDCl_3$): δ = 152.3 (151.8), 93.5 (93.1), 80.1 (79.4), 66.9 (66.6), 62.3 (62.0), 57.5 (57.3), 33.3, 32.4 (32.2), 28.4, 27.5 (26.7), 24.5 (23.2), 22.5 (22.4).

HRMS (QTOF ES+): *m/z* $[M + Na]^+$ calcd for $C_{14}H_{27}NNaO_4$:296.1831; found: 296.1838.

(*S*)-*tert*-Butyl 2,2-dimethyl-4-(pent-4-enyl)oxazolidine-3-carboxylate (9)

To a cold (−78 °C) stirred solution of oxalyl chloride (0.25 mL, 2.90 mmol) in anhydrous CH_2Cl_2 (10mL), anhydrous DMSO (0.41 mL, 5.19 mmol) was added over seven minutes under nitrogen atmosphere. The mixture was then allowed to warm to −60 °C over a period of 20 min. Then a solution of the alcohol **7** (500 mg, 1.92 mmol) in CH_2Cl_2 (3 mL) was added drop wise over 13 minutes. The mixture was warmed to -45 °C over 30 minutes. A solution of *N*-methylmorpholine (1.1 mL, 11.41 mmol), in CH_2Cl_2 (1 mL) was then added slowly. The mixture was allowed to warm to 0 °C and then transferred to a separating funnel charged with ice-cold 1 M HCl solution (25 mL) and CH_2Cl_2 (30 mL). The two phases were separated. The organic phase was then successively washed with water (50 mL) and brine (40 mL). Resulting organic layer was dried over $MgSO_4$ and concentrate under reduced pressure to give the crude aldehyde **8**, which was used for next step without purification.

The olefination of the aldehyde **8** was carried out as described in our earlier work [S1]. *n*-BuLi (2 M in hexane, 0.95 mL) was added dropwise over 10 min to a stirred suspension of the Wittig salt ($MePh_3P^+Br^-$) (696 mg, 1.94 mmol) in dry THF (2 mL) at 0 °C under argon atmosphere and the resulting solution was allowed to come to 10 °C over 30 min when the solution turned deep yellow. It was cooled to 0 °C and then a solution of the aldehyde **8** (350 mg, 1.29 mmol) in THF (5 mL) was added drop wise over 15 min while stirring. Stirring was continued at this condition for 30 min and then it was allowed to come to room temperature and further stirred for 3 h. The reaction mixture was then quenched with aqueous NH_4Cl solution (5 mL) and extracted with ethyl acetate (2 × 50 mL). The combined organic extract was washed successively with water (1×40 mL) and brine (1 × 40 mL), and then dried over $MgSO_4$. It was then filtered and the filtrate was concentrated in vacuo to leave

the crude product which was purified by flash chromatography over silica gel using a mixture of ethyl acetate-hexane (1:49) to give the product **9** as a colorless oil.

Yield: 371 mg, 72% over two steps.

$[\alpha]_D^{25}$ = +6.7 (*c* 1, $CHCl_3$);

IR ($CHCl_3$) 1980, 2937, 2871, 1698, 1642, 1458, 1390, 1376, 1258 cm^{-1};

^{1}H NMR (400 MHz, $CDCl_3$) δ = 5.75 (m, 1 H), 4.97-4.89 (m, 2 H), 3.87-3.83 (m, 2 H), 3.66 (m, 1 H), 2.02-2.00 (m, 2 H), 1.51 (s, 6 H), 1.48 (s, 2 H), 1.41 (s, 11 H).

^{13}C NMR (100 MHz, $CDCl_3$): δ = 152.1 (151.8), 138.3, 114.7 (114.5), 93.5 (93.0), 79.8 (79.3), 67.0 (66.7), 57.6 (57.2), 33.5 (33.1), 32.4, 28.4, 27.5 (26.7), 25.6 (25.5), 24.5 (23.2).

HRMS (QTOF ES+): *m/z* $(M + Na)^+$ calcd for $C_{15}H_{27}NNaO_3$: 292.1878 ; found: 292.1889.

(S)-2-(*tert*-Butoxycarbonylamino)hept-6-enoic acid (10)

The oxidation was carried out in a similar manner as described by us [S1]. An aqueous solution of chromic acid (8 mL, 8 equiv) was added dropwise to a vigorously stirred solution of **9** (550 mg, 2.06 mmol) in acetone (10 mL) and stirring was continued for 2 h at rt. The reaction mixture was then quenched by isopropyl alcohol and the acetone was evaporated under reduced pressure. The resulting mixture was then poured into water (40 mL) and extracted with ether (3 × 25 mL).The organic layer was washed successively with water (3 × 20 mL), brine (30 mL) and then dried over $MgSO_4$. It was filtered and the filtrate was concentrate to leave the crude product as a yellowish liquid, which on chromatographic purification over silica using petroleum ether/ethyl acetate (1:1) as eluent afforded the product **10** as a colorless liquid.

Yield: 365 mg, 73%.

$[\alpha]_D^{25}$ = +13.9 (*c* 1.5, $CHCl_3$); Lit. + 11.5 (*c* 1, $CHCl_3$) [15].

IR ($CHCl_3$) 3346, 2980, 2932, 1718, 1511, 1396, 1369, 1166 cm^{-1};

^{1}H NMR (400 MHz, $CDCl_3$) δ = 6.12 (br s, 1 H), 5.71 (m, 1 H), 4.95 (d, 1H, J = 17.2 Hz), 4.91 (d, 1H, J = 11.6 Hz), 4.24 (br s, 1H), 2.03-1.99 (m, 2H), 1.80 (br s, 1H), 1.50 (br s, 1H), 1.43 (merged singlet, 11H).

^{13}C NMR (100 MHz, $CDCl_3$): δ = 177.5, 155.6, 137.9, 115.1, 80.2, 53.3, 33.2, 31.9, 28.3, 24.5.

HRMS (QTOF ES+): *m/z* $(M + Na)^+$ calcd for $C_{12}H_{21}NNaO_4$:266.1350; found: 266.1368.

(*S*)-Methyl 2-(*tert*-butoxycarbonylamino)hept-6-enoate (11)

Methyl iodide (0.16 mL, 2.6 mmol) was added dropwise to a stirred solution of **10** (250 mg, 1.03 mmol) in dry DMF (6 mL) at 0 °C under nitrogen atmosphere in presence of anhydrous Cs_2CO_3 (550 mg, 1.68 mmol) and stirring continued for 2h at room temperature. It was then diluted with water (50 mL) and extracted with ether (2 × 50 mL). The combined organic layer was washed successively with water (2 × 75 mL) and brine (75 mL). The organic extract was then dried over anhydrous Na_2SO_4, filtered and the filtrate was concentrated under reduced pressure to leave a crude product, which was purified by column chromatography on silica gel using ethyl acetate in petroleum ether (1:49) solvent provided the compound **11** as a colorless liquid.

Yield: 232 mg, 88%

$[\alpha]_D^{25}$ = +14.0 (*c* 0.5, $CHCl_3$); *Lit*. + 14.6 (*c* 1, $CHCl_3$) [15].

IR ($CHCl_3$) 3373, 3078, 2978, 2931, 1743, 1714, 1505, 1367, 1164 cm^{-1};

^{1}H NMR (400 MHz, $CDCl_3$) δ = 5.70 (m, 1H), 4.96-4.91 (m, 2H), 4.89 (s, 1H), 4.23 (m, 1H), 3.67 (s, 3H), 2.03-1.98 (m, 2H), 1.70 (m, 1H), 1.56 (m, 1H), 1.42-1.34 (m, 11H).

^{13}C NMR (100 MHz, $CDCl_3$): δ = 173.4, 155.4, 137.9, 115.0, 79.8, 53.3, 52.2, 33.1, 32.1, 28.3, 24.5.

HRMS (QTOF ES+): *m/z* $(M + Na)^+$ calcd for $C_{13}H_{23}NNaO_4$: 280.1519; found: 280.1531.

(*S*,*E*)-1-Benzyl 8-methyl 7-(*tert*-butoxycarbonylamino)oct-2-enedioate (14b)

This was prepared following the general procedure and the product was obtained as a colorless liquid. Yield: 175 mg, 90%

$[\alpha]_D^{25}$ = +12.7 (*c* 1.50, $CHCl_3$).

IR (neat): Ṽ = 3366, 2977, 2953, 2869, 1726, 1714, 1654, 1505, 1455, 1368, 1264, 1164 cm^{-1}.

^{1}H NMR (400 MHz, $CDCl_3$): δ = 7.37-7.32 (m, 5 H), 6.96 (td, *J* = 6.8, 15.6 Hz, 1 H), 5.87 (d, *J* = 15.6 Hz, 1 H), 5.17 (s, 2 H), 5.07 (d, *J* = 8 Hz, 1 H), 4.31 (m, 1 H), 3.73 (s, 3 H), 2.26-2.20 (m, 2 H), 1.86-1.80 (m, 2 H), 1.68-1.51 (m, 2 H), 1.44 (s, 9 H) ppm.

^{13}C NMR (100 MHz, $CDCl_3$): δ = 173.1, 166.3, 155.4, 148.8, 136.1, 128.5, 128.2, 121.6, 79.9, 66.1, 53.1, 52.3, 32.3, 31.6, 28.3, 23.8 ppm.

HRMS (TOF MS ES+): *m/z* $[M + Na]^+$ calcd. for $C_{21}H_{29}NNaO_6$ 414.1893; found 414.1874.

(*S*)-Methyl 2-(*tert*-butoxycarbonylamino)-8-oxo-8-(phenylamino)octanoate (14c)

This was prepared following the general procedure and the product was obtained as a colorless liquid. Yield: 165 mg, 88%.

$[\alpha]_D^{25}$ = −66.0 (*c* 0.5, MeOH)

IR (KBr, cm^{-1}): 3776, 3301, 3132, 3059, 2923, 2852, 1742, 1647, 1598, 1538, 1498, 1443, 1368, 1340, 1246, 1161, 1077, 1028, 1003, 990, 975, 963, 905, 858, 798, 751;

^{1}H NMR (DMSO-d_6, 400MHz) : δ = 9.82 (s, 1H), 7.63 (d, *J* = 8.0 Hz, 2H), 7.26-7.17 (m, 3H), 7.03 (d, *J* = 6.4Hz, 1H), 6.74 (td, *J* = 15.2, 6.4 Hz, 1H), 6.05 (d, *J* = 15.2 Hz, 1H), 3.95 (m, 1H), 3.48 (s, 3H), 2.16-2.03 (m, 2H), 1.66-1.57 (m, 2H), 1.47-1.43 (m, 2H), 1.34 (s, 9H) ppm;

^{13}C NMR ($CDCl_3$, 400MHz): δ = 173.5, 164.1, 156.0, 144.2, 139.6, 128.9, 125.3, 123.5, 119.7, 53.7, 52.0, 31.3, 30.8, 28.6, 24.7 ppm.

HRMS (TOF MS ES+): *m/z* $[M + Na]^+$ calcd. for $C_{20}H_{28}N_2NaO_5$ 399.1896; found 399.1884.

General procedure of hydrogenation

This was carried out in a similar manner as described in [12]. Pd/C (30 mg) was added to a stirring solution of cross metathesis product **14a/14b/14c** (0.50 mmol) in dry MeOH (5 mL). The resulting mixture was then stirred for 2 h at room temperature under hygrogen atmosphere. The reaction mixture was then filtered through celite, the filter cake was washed with methanol (5 mL) and the combined filtrate was concentrated in vacuo. The residue thus obtained was subjected to column chromatographic purification over silica gel using appropriate mixture of ethyl acetate in hexane to provide the hydrogenated product **15a–c** respectively as colorless viscous liquids.

(S)-8-*tert*-Butyl 1-methyl 2-(*tert*-butoxycarbonylamino)octanedioate (15a)

Yield: 79%

$[\alpha]_D^{25}$ = +8.45 (*c* 2.00, $CHCl_3$).

IR (neat): 3375, 2978**,** 2932, 2863, 1729, 1715, 1506, 1367 cm^{-1}.

^{1}H NMR (400 MHz, $CDCl_3$): δ 5.03 (d, *J* = 8 Hz, 1 H), 4.28 (m, 1 H), 3.74 (s, 3 H), 2.19 (t, J = 7.2 Hz, 2 H), 1.78 (m, 1 H), 1.61-1.54 (m, 3 H), 1.44 (overlapping singlet, 18 H), 1.35-1.33 (m, 4 H).

^{13}C NMR (100 MHz, $CDCl_3$): δ 173.4, 173.1, 155.4, 80.0, 79.8, 53.3, 52.2, 35.3, 32.6, 28.6, 28.3, 28.1, 24.9, 24.8.

HRMS (TOF MS ES+): m/z $[M + Na]^+$calcd. for $C_{18}H_{33}NNaO_6$: 382.2206; Found: 382.2214.

(S)-7-(*tert*-Butoxycarbonylamino)-8-methoxy-8-oxooctanoic acid (15b)

Yield: 82%

$[\alpha]_D^{25}$ = - 14.67 (*c* 1.50, MeOH); Lit. -13.5 (*c* 1.0, MeOH). [35]

IR (neat): 3334, 2935, 2864, 1738, 1722, 1715, 1515, 1436, 1368, 1167 cm^{-1}.

^{1}H NMR (400 MHz, $CDCl_3$): δ 5.10 (d, *J* = 8.4Hz, 1H), 4.29 (m, 1H), 3.74 (s, 3H), 2.34 (t, *J* = 7.2 Hz, 2H), 1.78 (m, 1H), 1.65-1.60 (m, 3H), 1.44 (s, 9H), 1.37-1.33 (m, 4H).

^{13}C NMR (100 MHz, $CDCl_3$): δ 179.1, 173.5, 155.5, 79.9, 53.3, 52.3, 33.8, 32.5, 28.6, 28.3, 24.9, 24.4.

HRMS (TOF MS ES+): m/z $[M + Na]^+$calcd. for $C_{14}H_{25}NNaO_6$: 326.1580; Found: 326.1592.

Methyl 2-(*tert*-butoxycarbonylamino)-8-oxo-8-(phenylamino)octanoate (15c)

Yield: 90%

$[\alpha]_D^{25}$ = +1.60 (c 1.5, $CHCl_3$)

IR (KBr, cm^{-1}): 3850, 3747, 3320, 2927, 2855, 1744, 1694, 51, 1210, 1163, 1049, 1020, 867, 756.

^{1}H NMR ($CDCl_3$, 400MHz) : δ = 7.76 (s, 1H), 7.47 (d, *J* = 8.0 Hz, 2H), 7.31-7.20 (m, 3H), 5.04 (d, *J* = 7.6 Hz, 1H), 4.21 (d, *J* = 4.8 Hz, 1H), 3.65 (s, 3H), 2.25 (t, *J* = 7.2 Hz, 2H), 1.69-1.50 (m, 6H), 1.37 (s, 9H), 1.30-1.18 (m, 2H) ppm.

^{13}C NMR ($CDCl_3$, 400MHz): δ = 173.4, 171.6, 155.6, 138.1, 128.9, 124.1, 119.8, 80.1, 53.2, 52.3, 37.2, 32.7, 29.7, 28.3, 25.3, 24.9 ppm.

HRMS (TOF MS ES+): *m/z* $[M + Na]^+$ calcd. for $C_{20}H_{30}N_2NaO_5$ 401.2052; found 401.2038.

Reference

S1 Pahari, A. K.; Mukherjee, J. P.; Chattopadhyay, S. K. *Tetrahedron* **2014,** *70,* 7185–7191. doi:10.1016/j.tet.2014.07.045

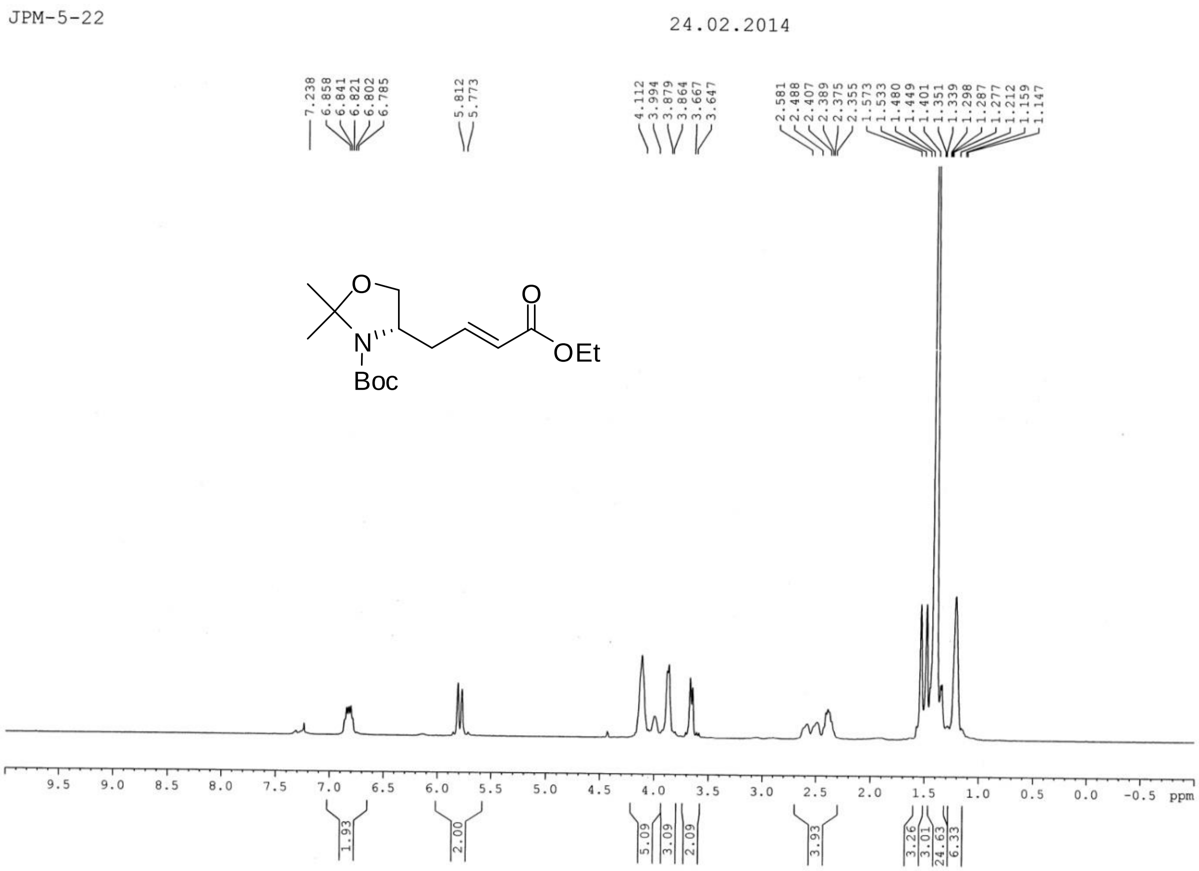

^{1}H NMR of compound **5** in $CDCl_3$

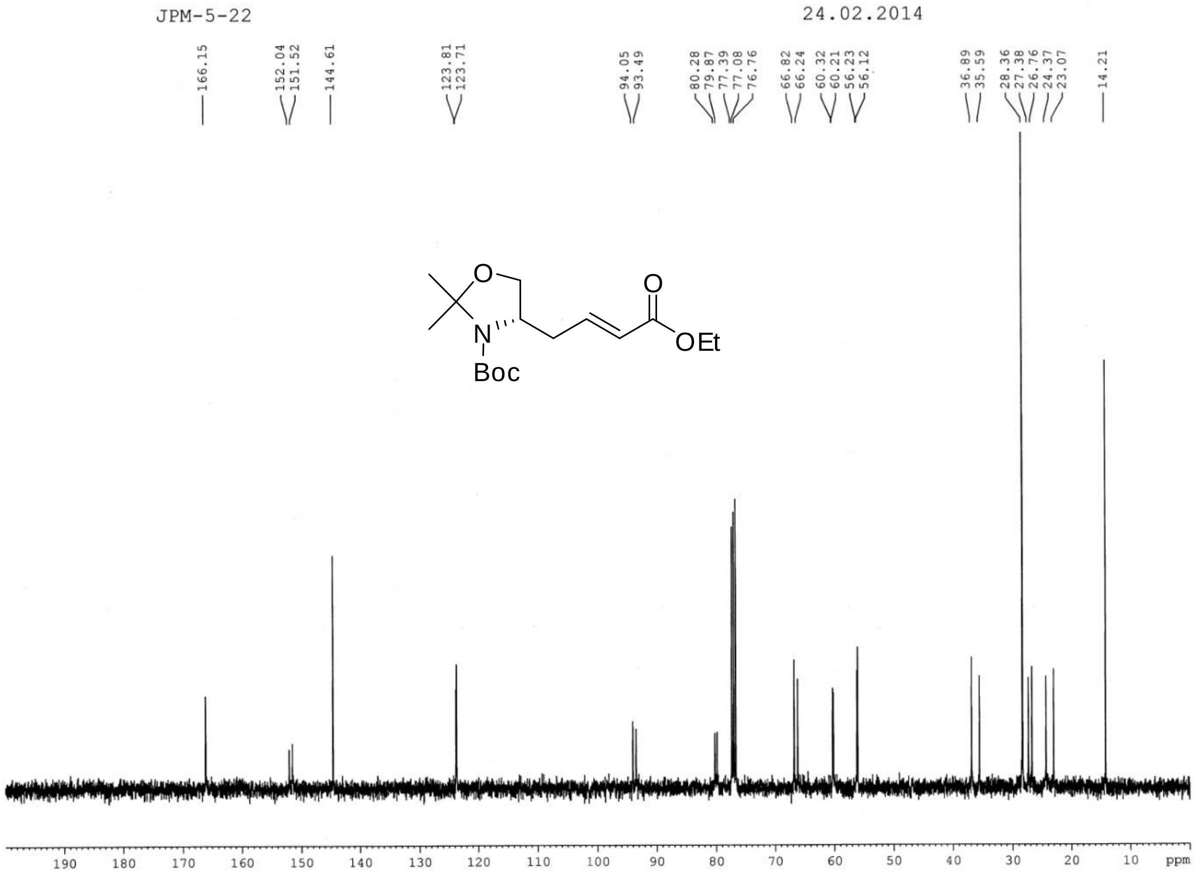

^{13}C NMR of compound **5** in $CDCl_3$

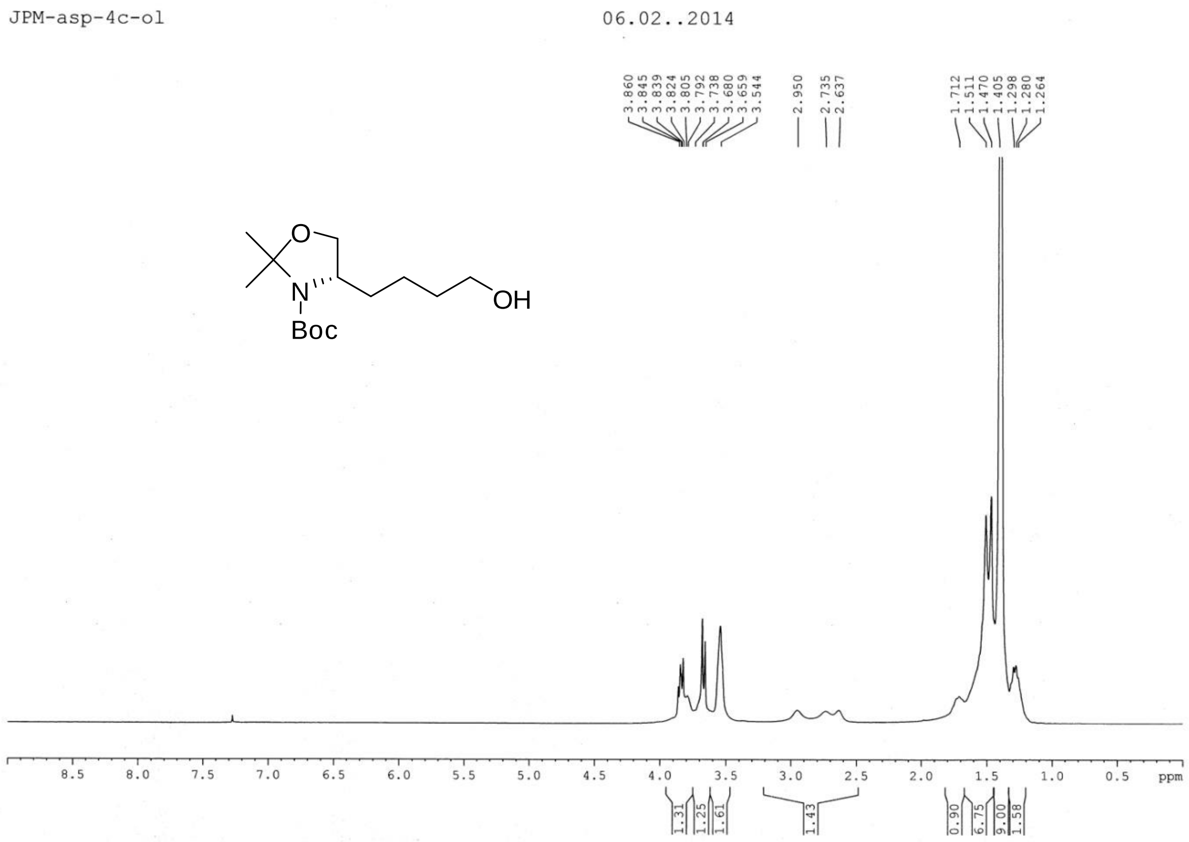

^{1}H NMR of compound **7** in $CDCl_3$

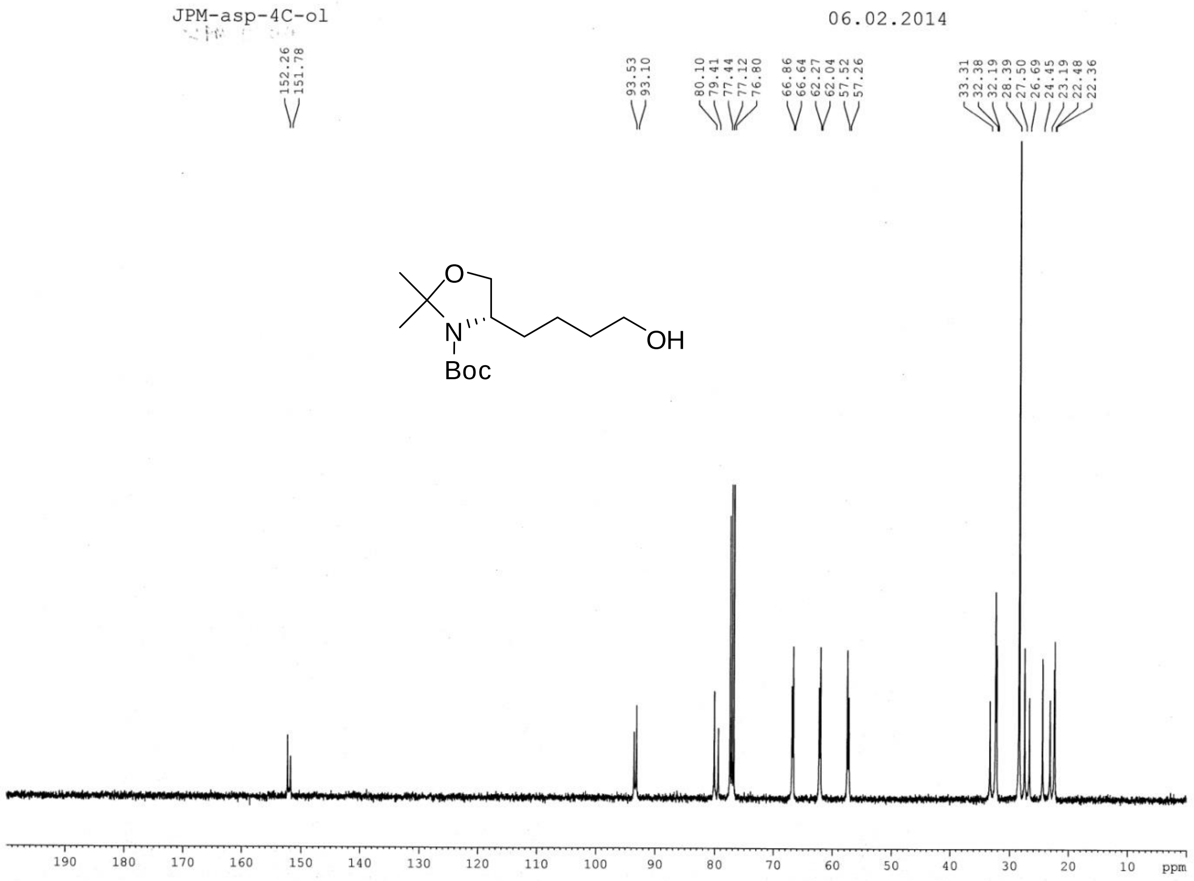

^{13}C NMR of compound **7** in $CDCl_3$

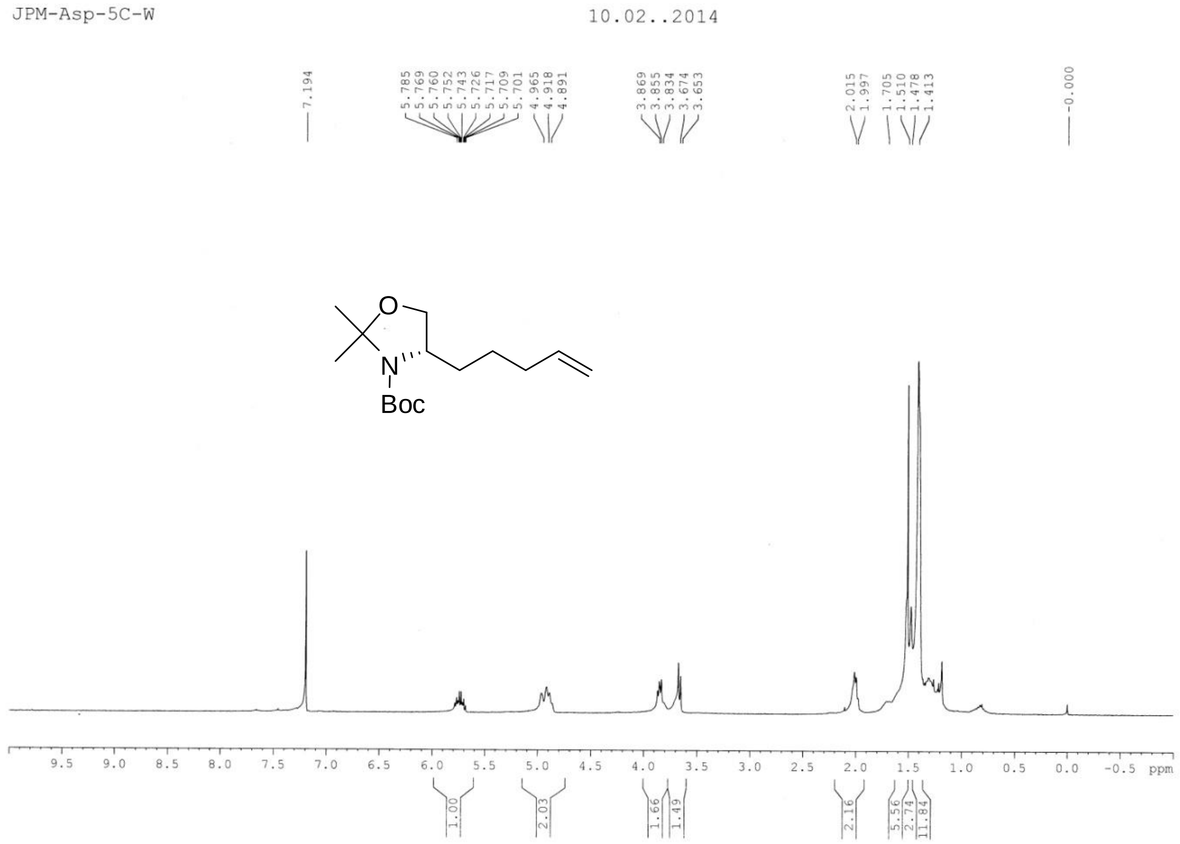

^{1}H NMR of compound **9** in $CDCl_3$

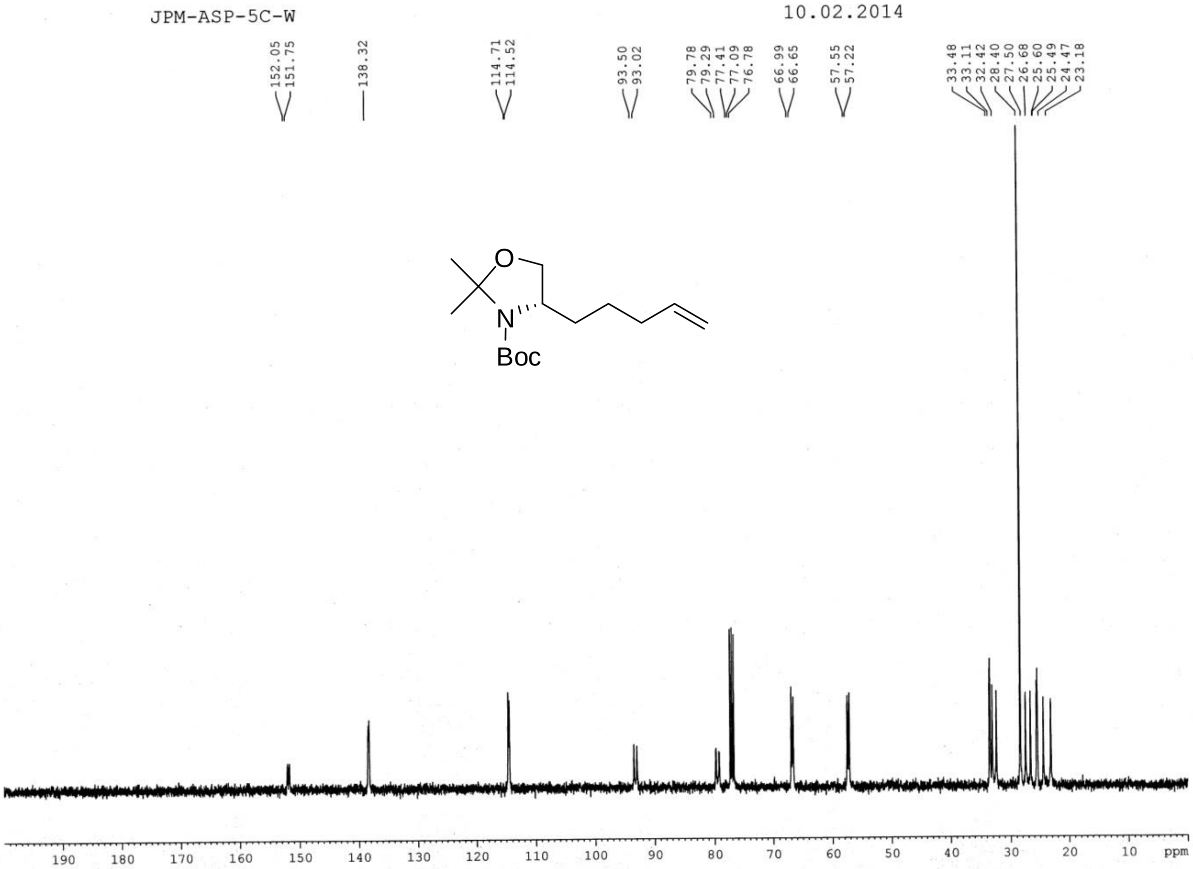

^{13}C NMR of compound **9** in $CDCl_3$

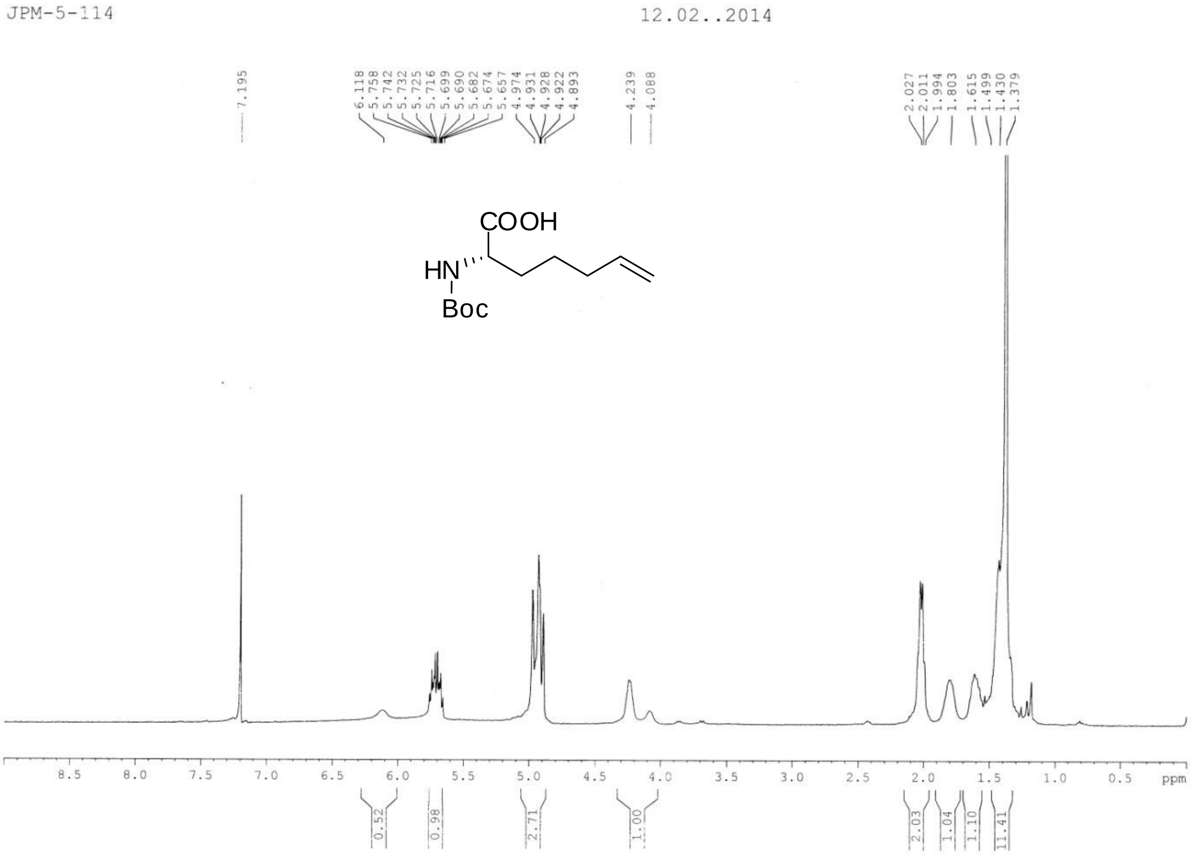

^{1}H NMR of compound **10** in $CDCl_3$

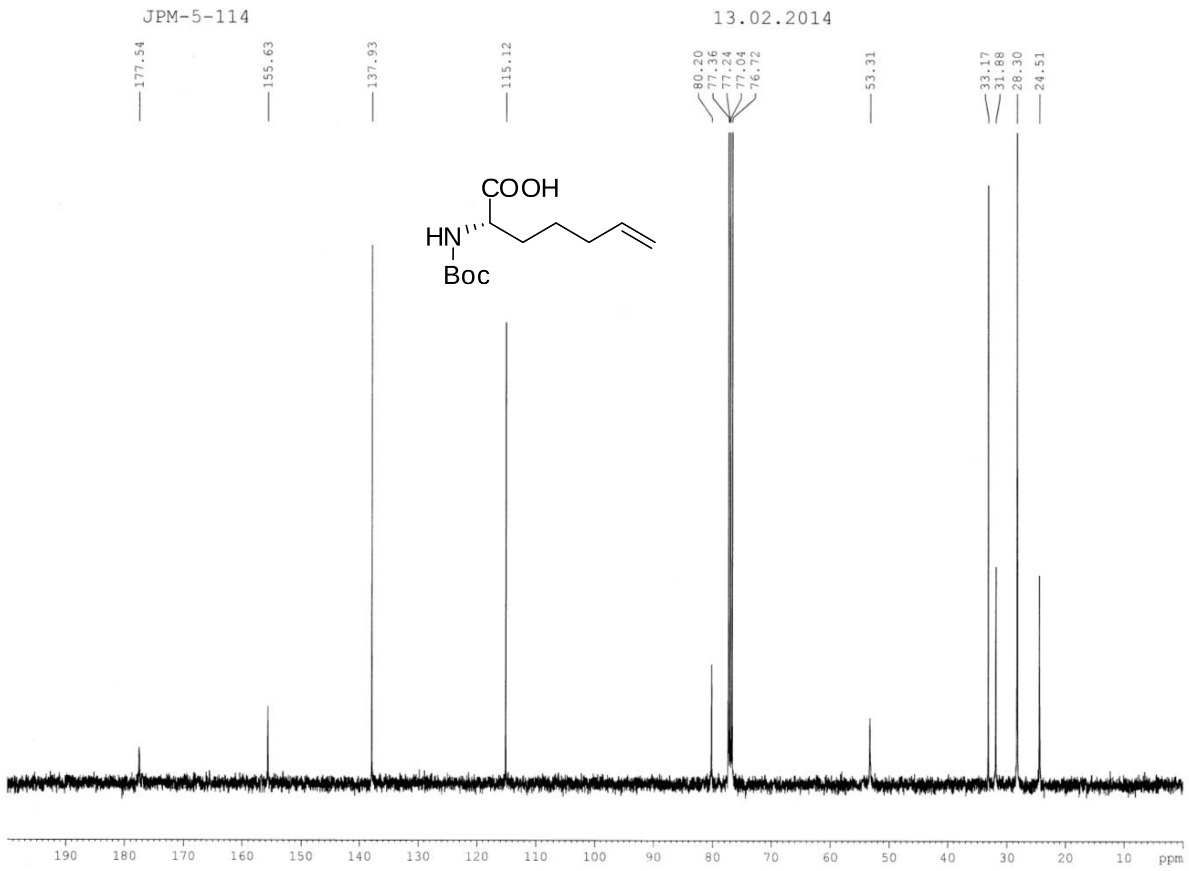

^{13}C NMR of compound **10** in $CDCl_3$

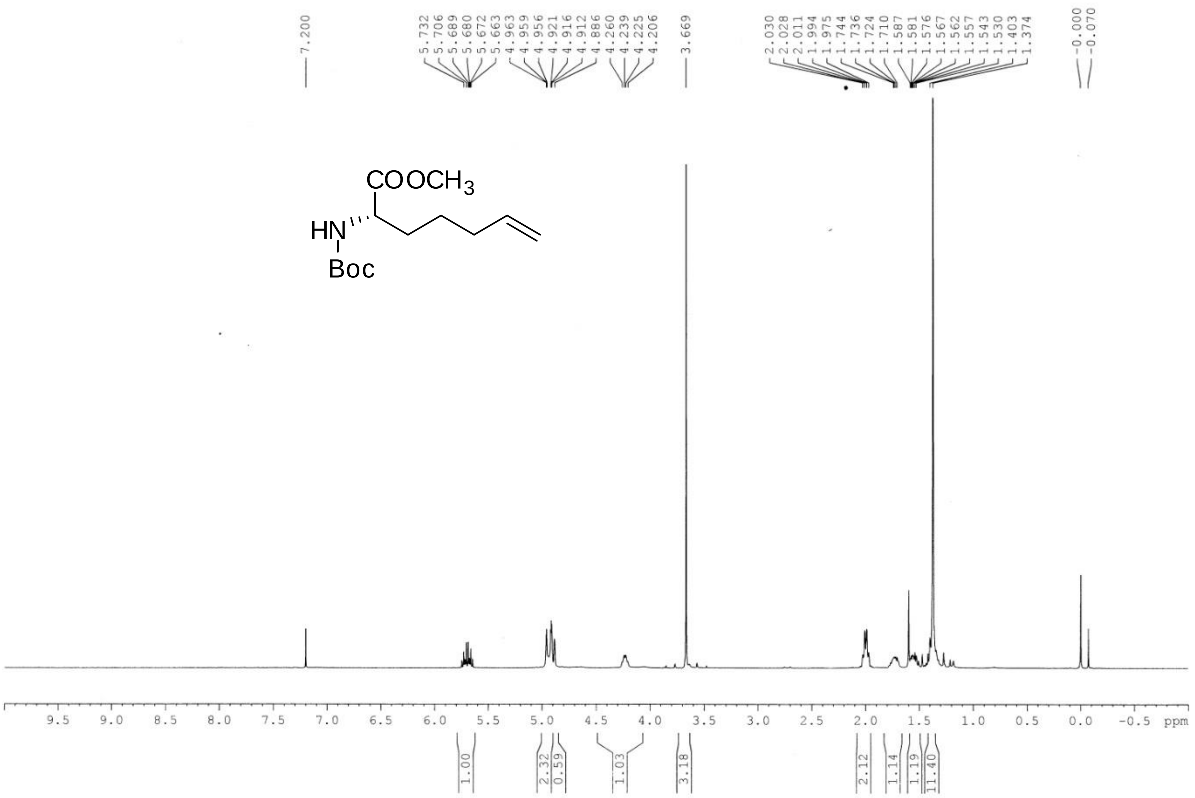

^{1}H NMR of compound **11** in $CDCl_3$

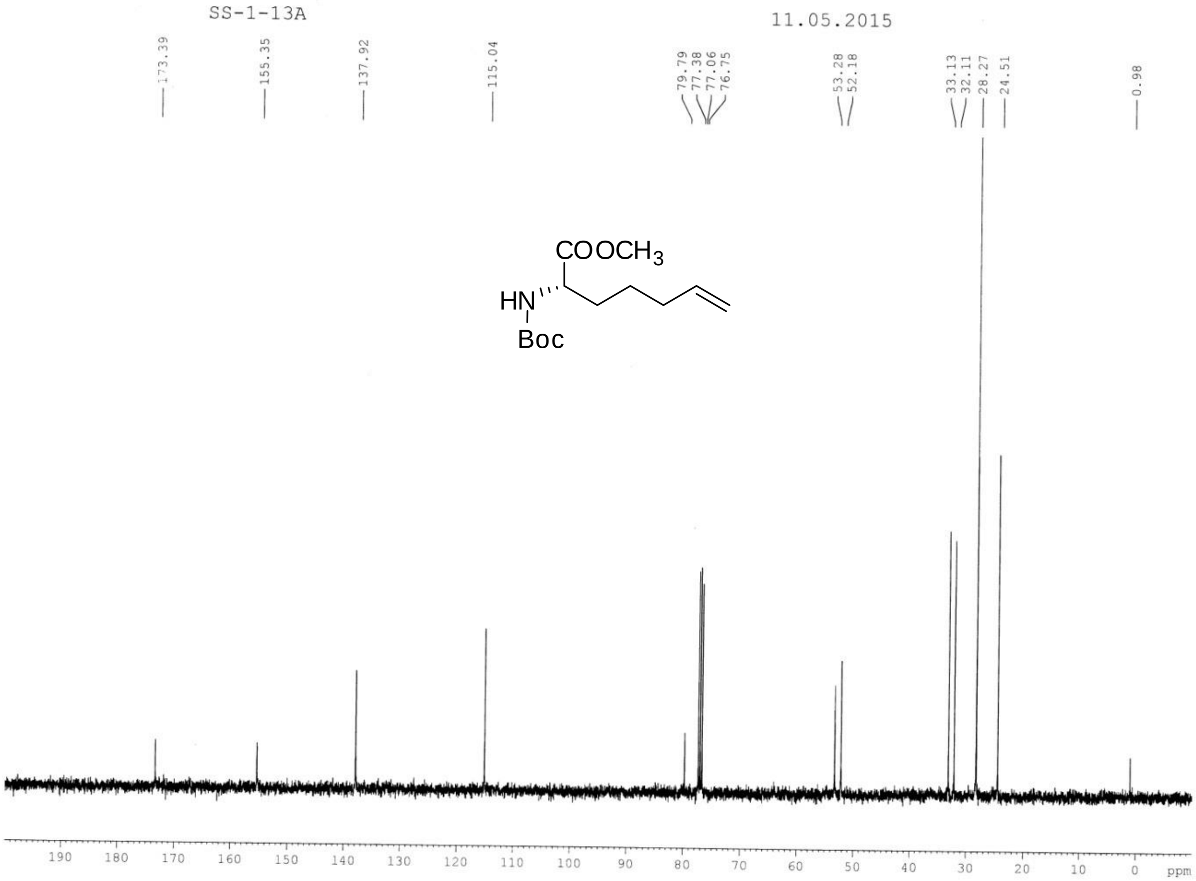

^{13}C NMR of compound **11** in $CDCl_3$

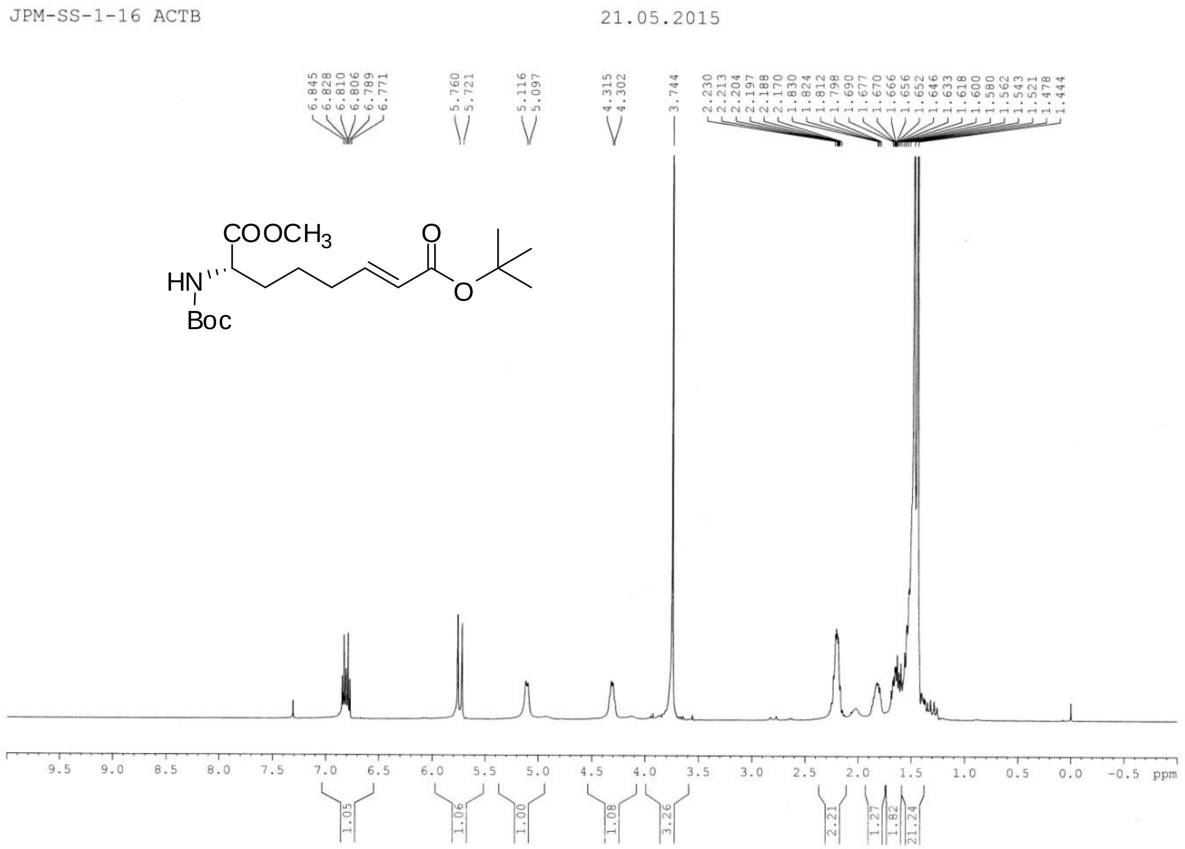

^{1}H NMR of compound **14a** in $CDCl_3$

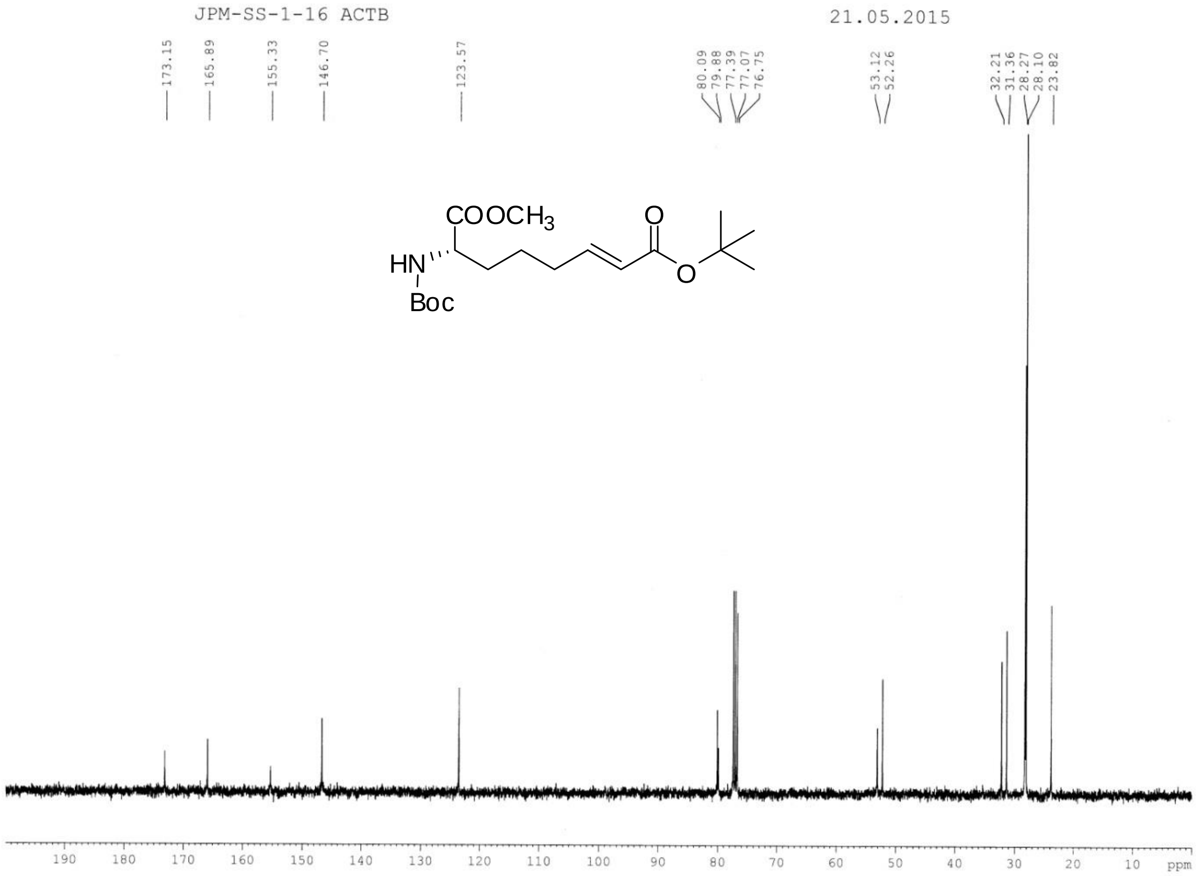

^{13}C NMR of compound **14a** in $CDCl_3$

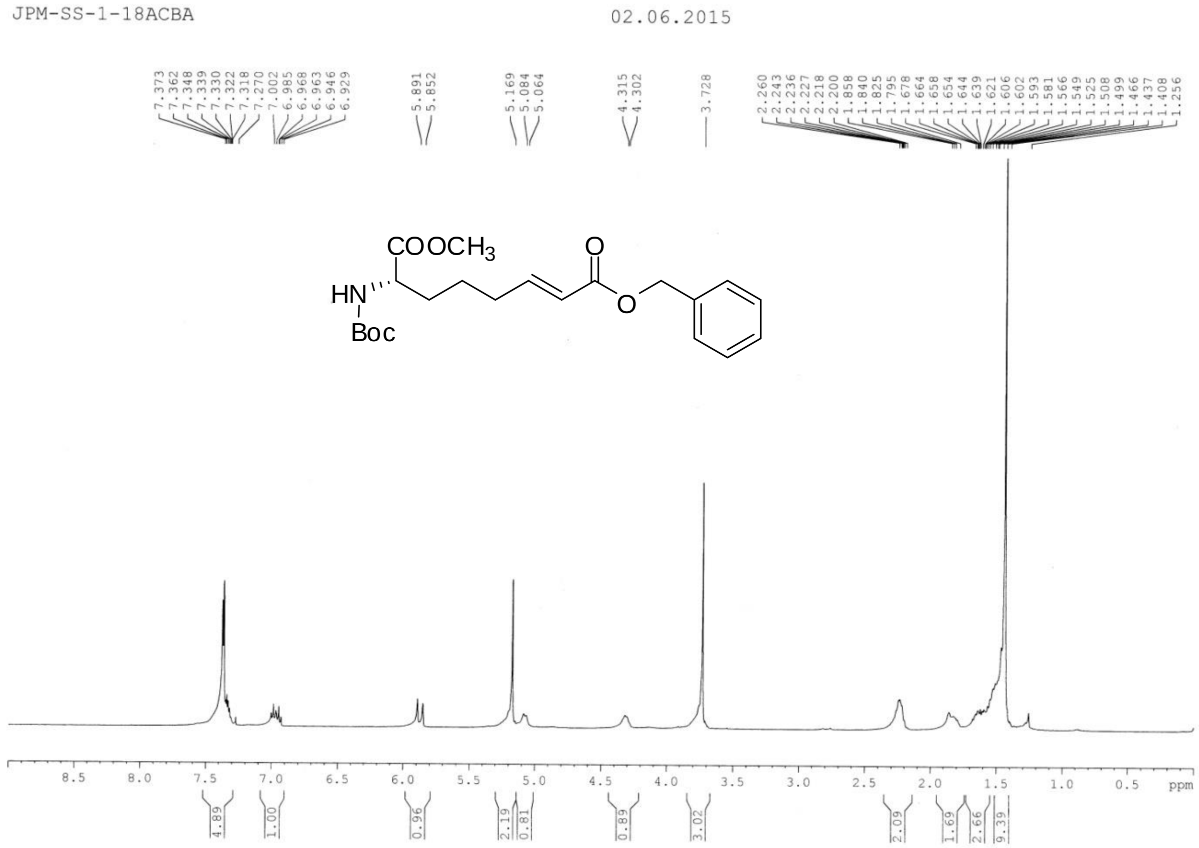

^{1}H NMR of compound **14b** in $CDCl_3$

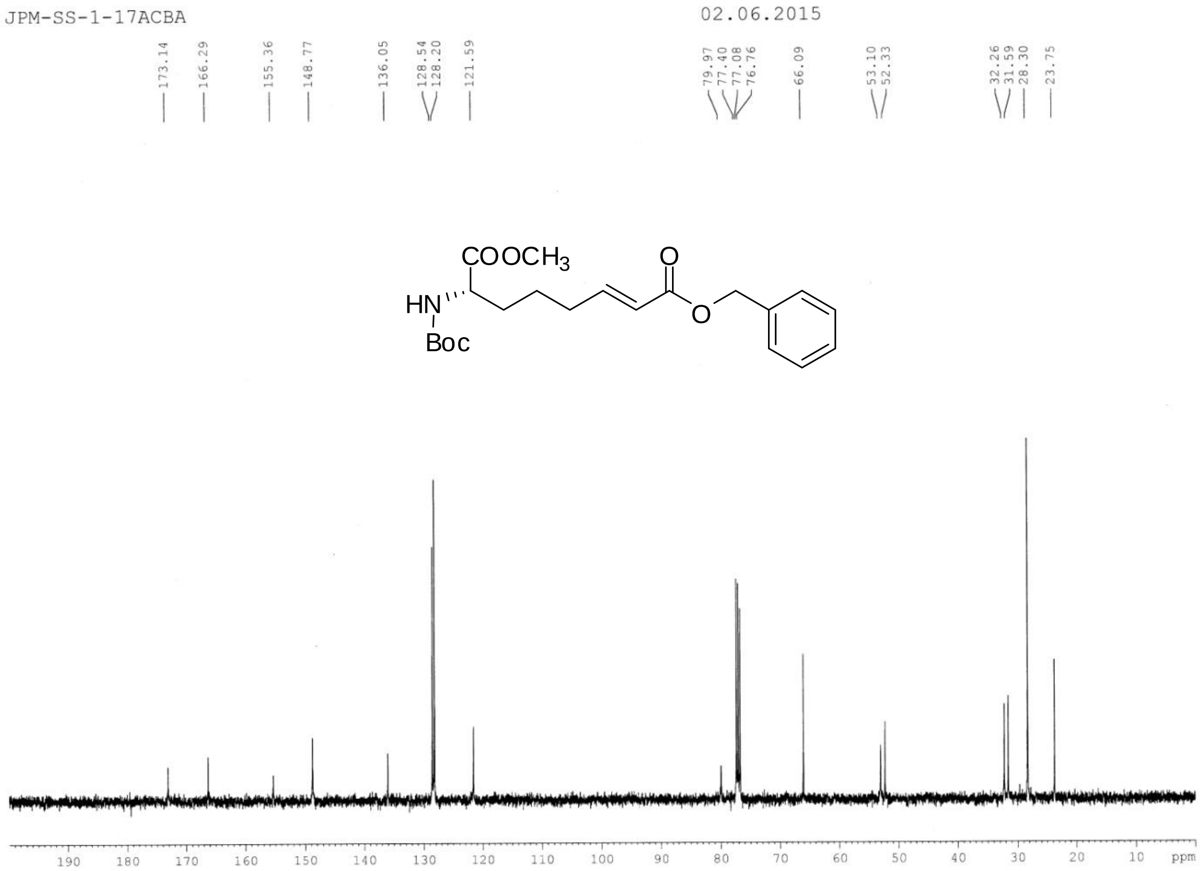

^{13}C NMR of compound **14b** in $CDCl_3$

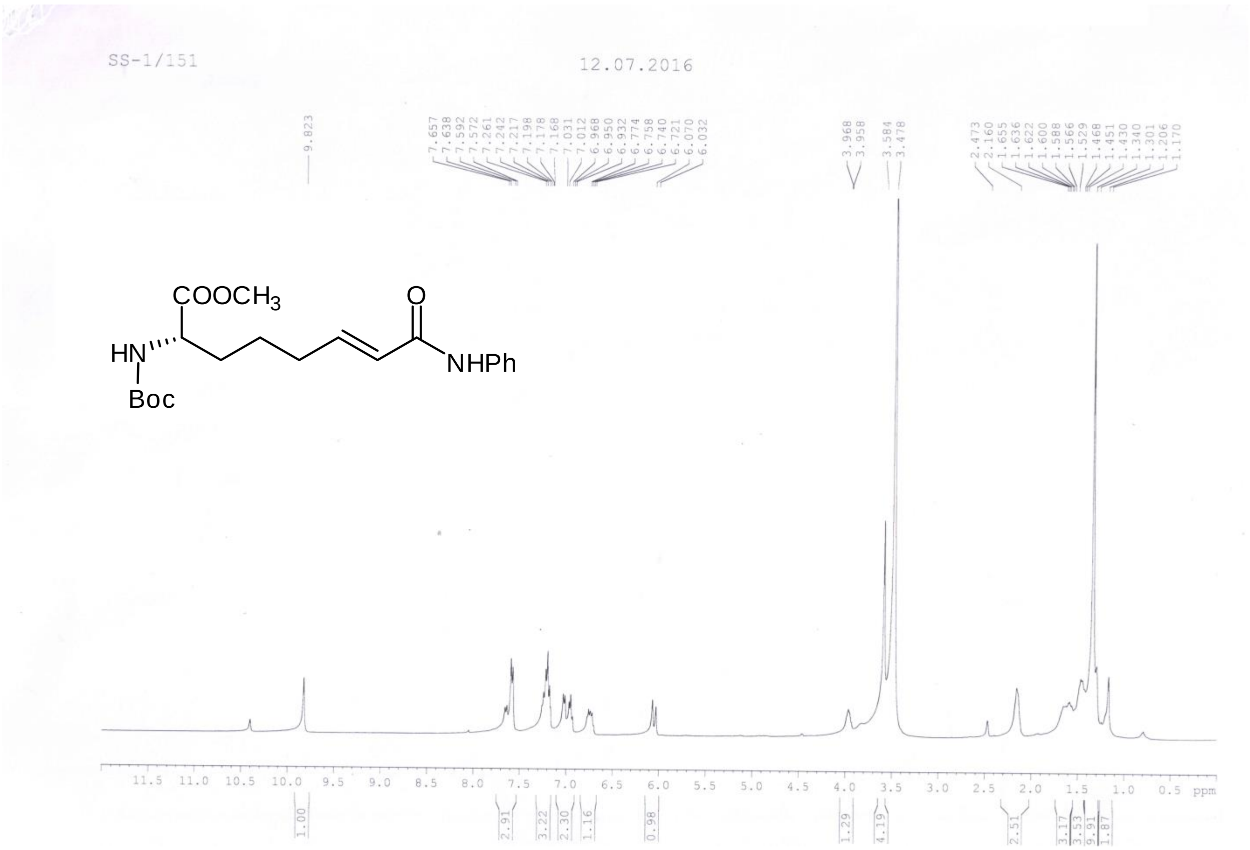

^{1}H NMR of compound **14c** in $CDCl_3$

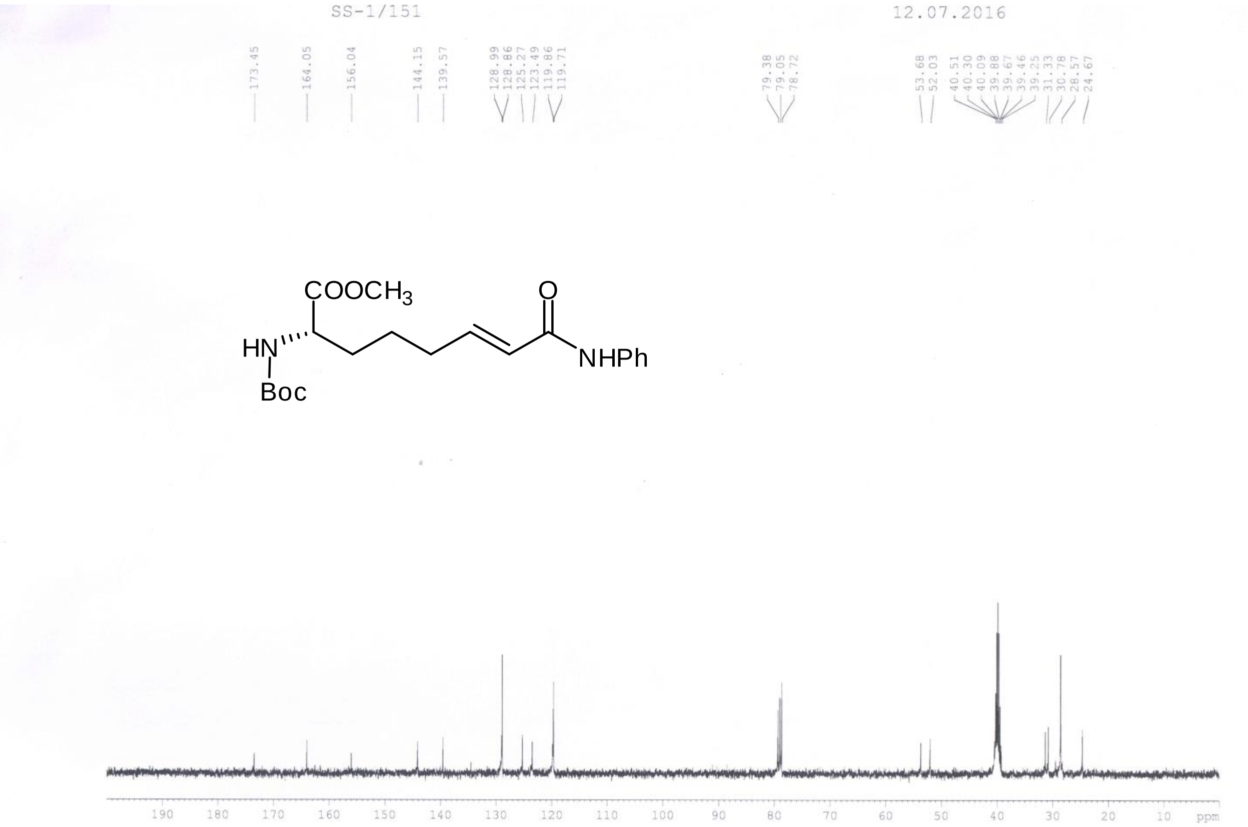

^{13}C NMR of compound **14c** in $CDCl_3$

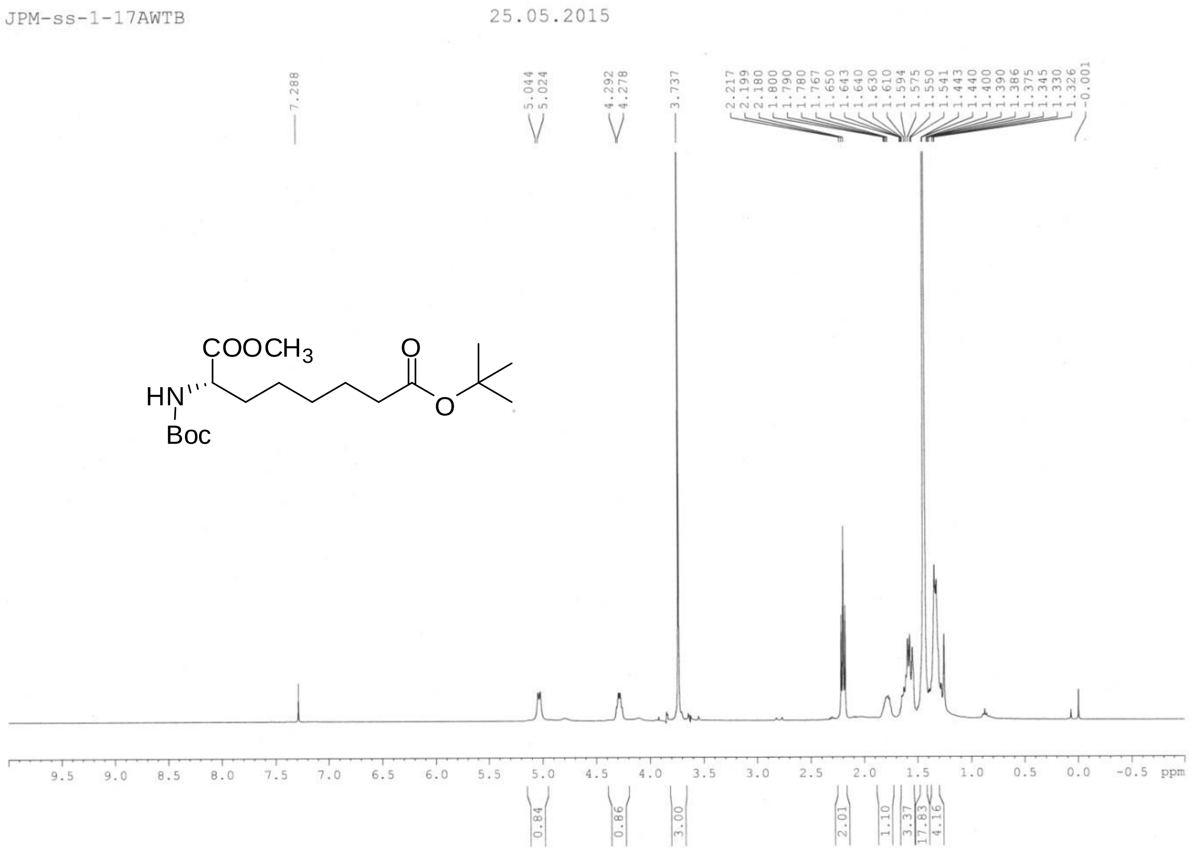

^{1}H NMR of compound **15a** in $CDCl_3$

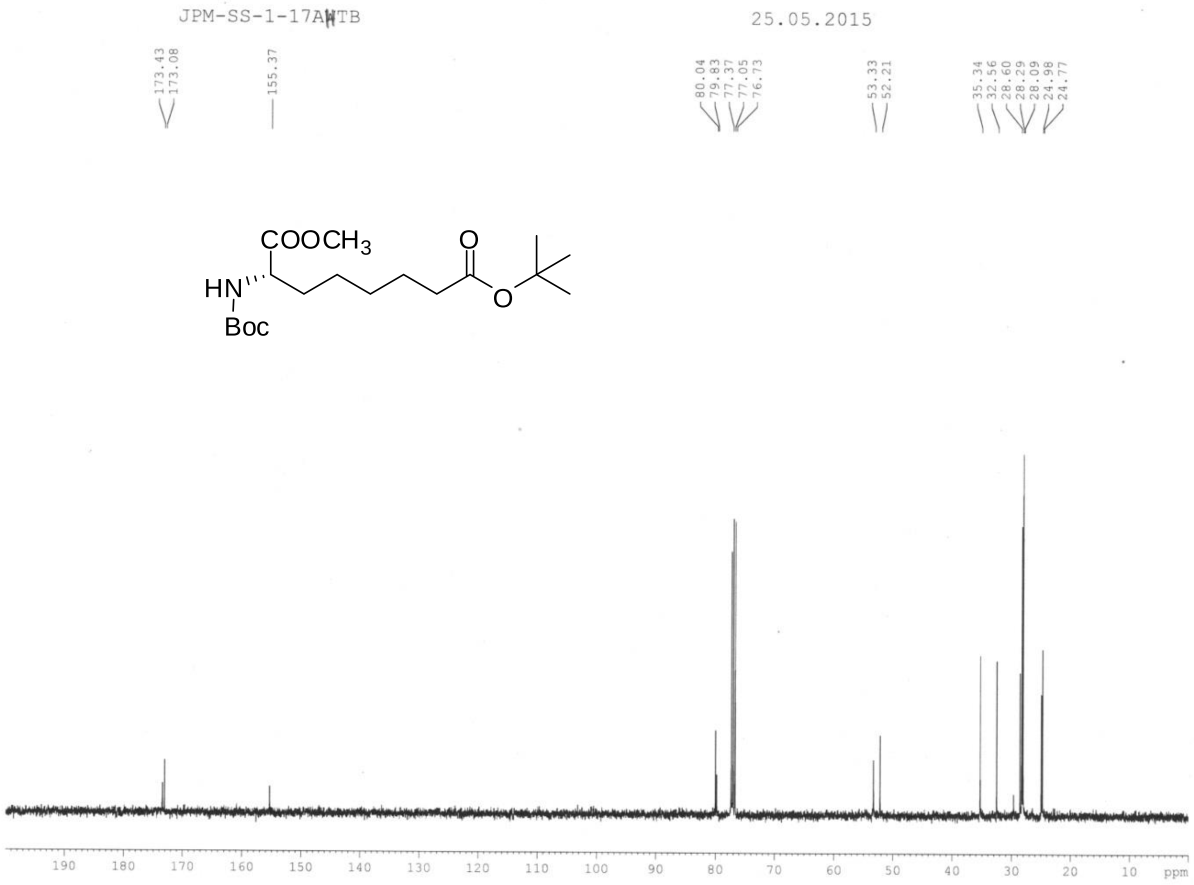

^{13}C NMR of compound **15a** in $CDCl_3$

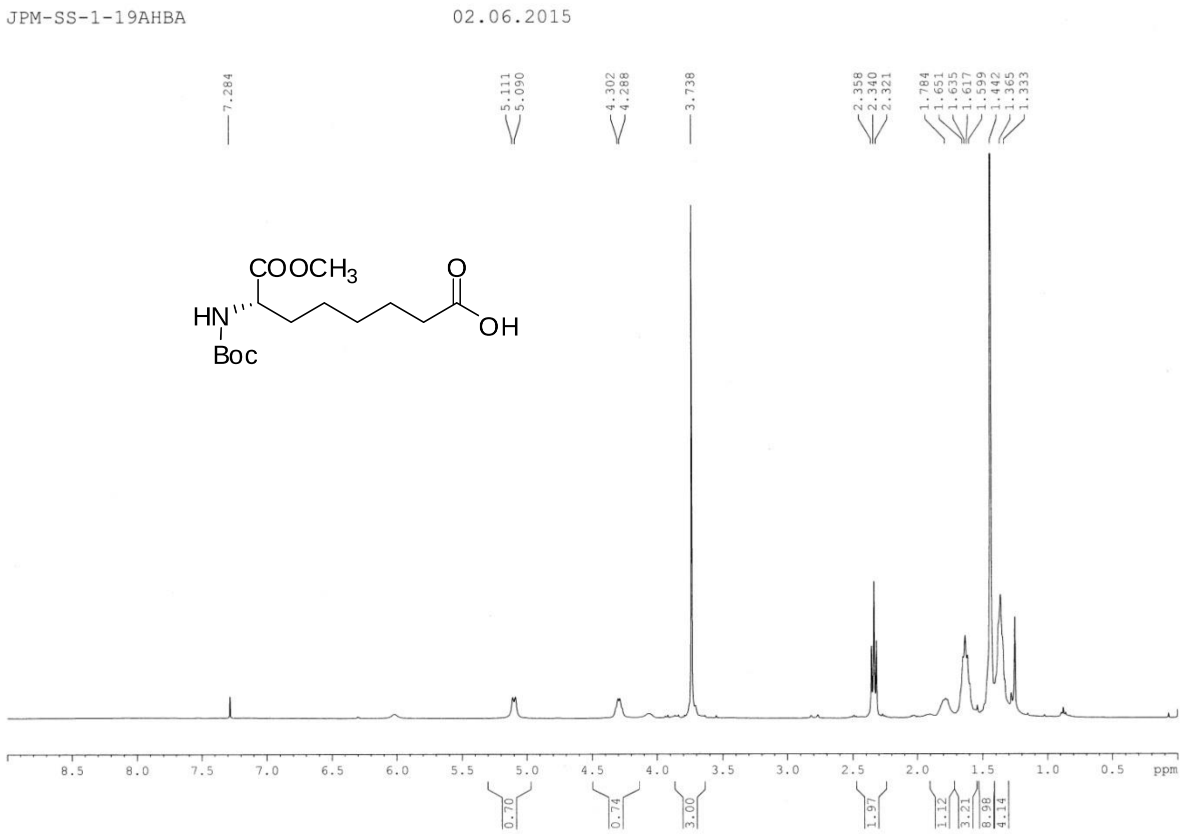

^{1}H NMR of compound **15b** in $CDCl_3$

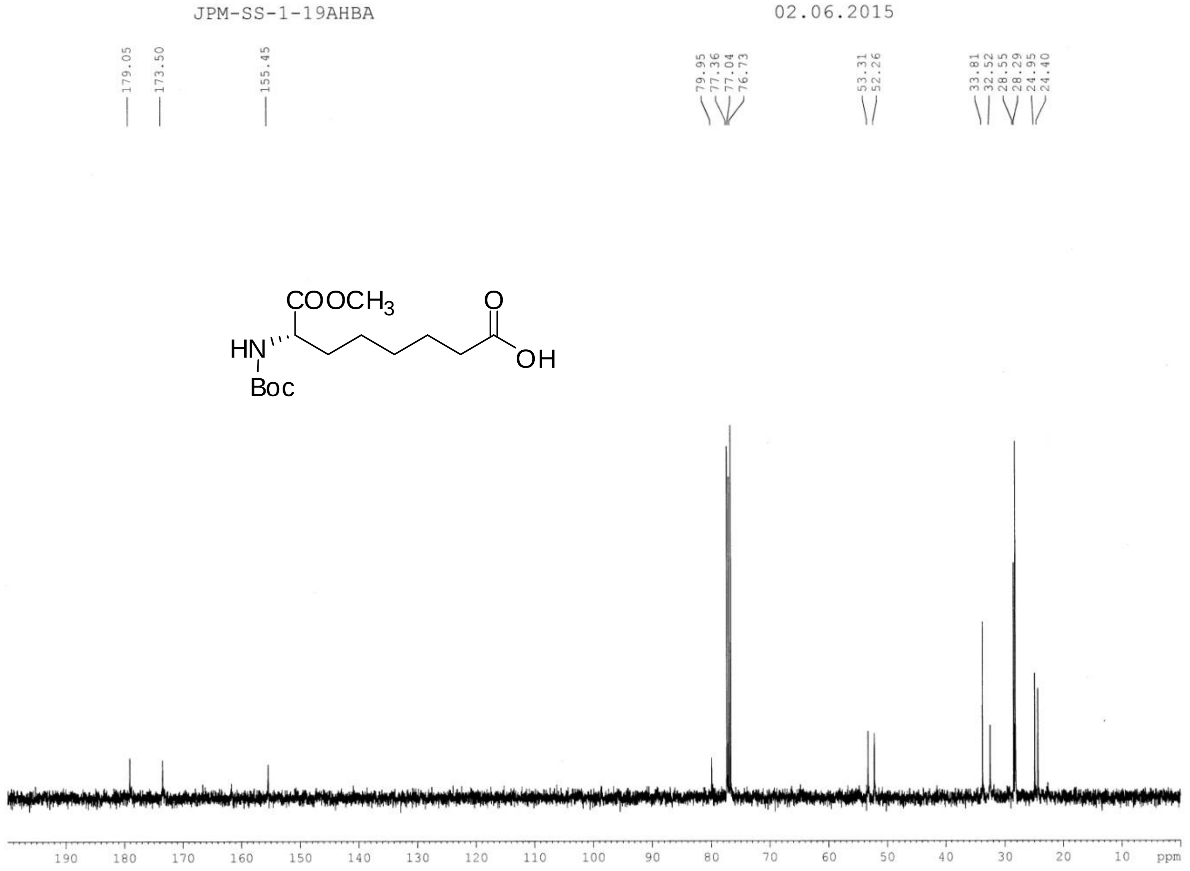

^{13}C NMR of compound **15b** in $CDCl_3$

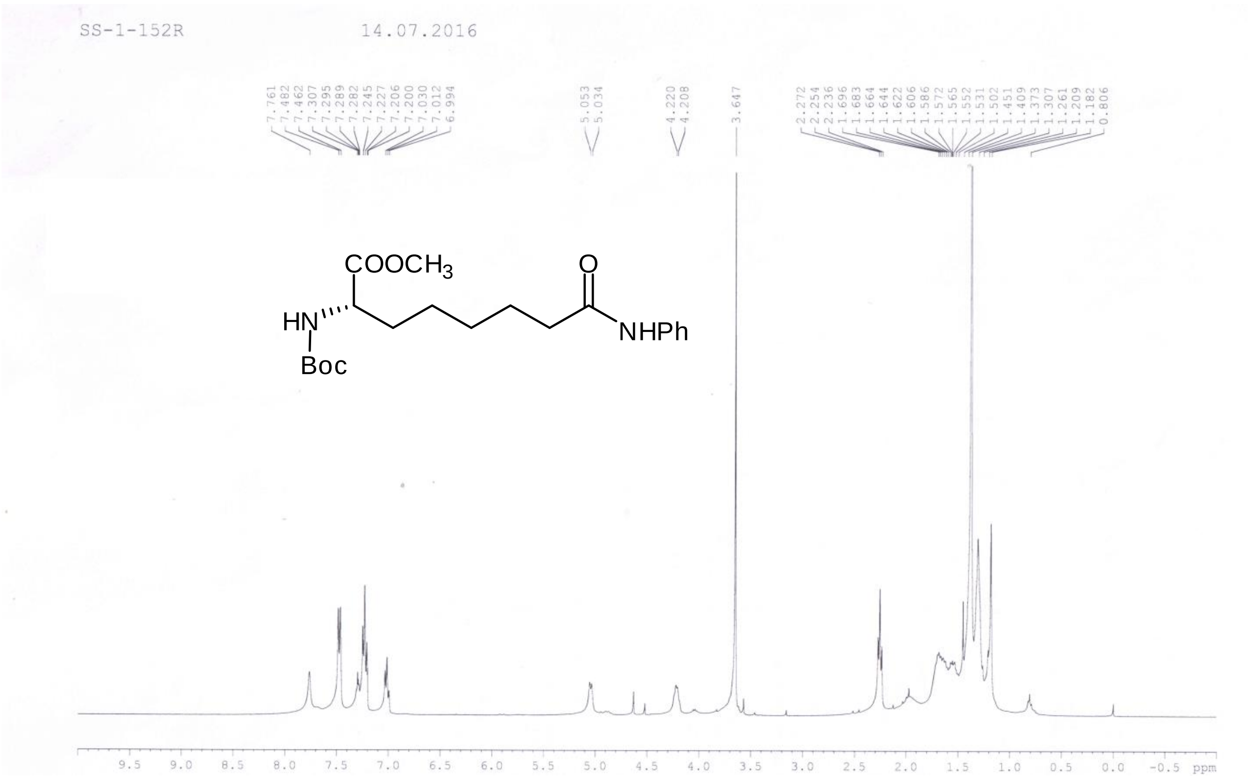

^{1}H NMR of compound **15c** in $CDCl_3$

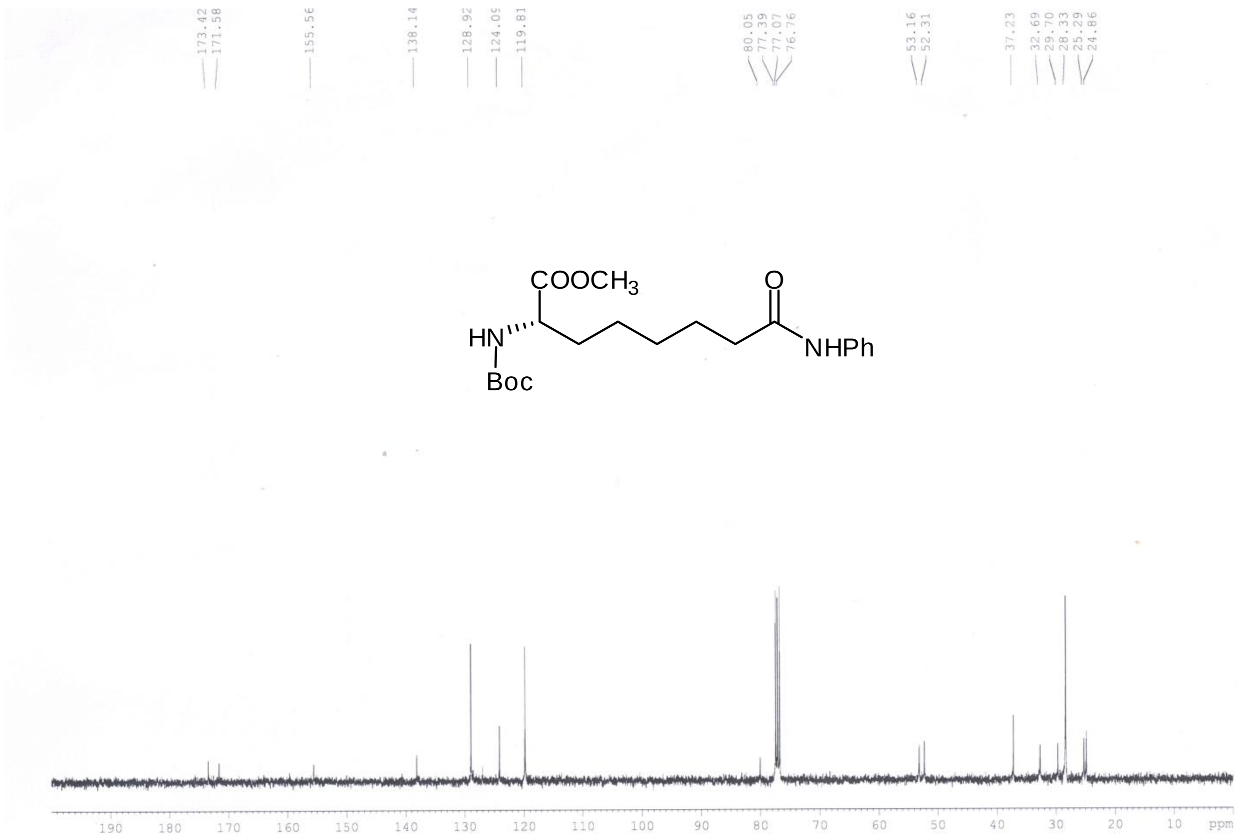

^{13}C NMR of compound **15c** in $CDCl_3$
